# The Role of Mammalian Creb3-Like Transcription Factors in Response to Nutrients

**DOI:** 10.3389/fgene.2019.00591

**Published:** 2019-06-21

**Authors:** Haris A. Khan, Carla E. Margulies

**Affiliations:** Physiological Chemistry, Biomedical Center, Ludwig-Maximilians-Universität, Munich, Germany

**Keywords:** Creb3, metabolism, transcription, chromatin, secretion, metabolic disease

## Abstract

Our ability to overcome the challenges behind metabolic disorders will require a detailed understanding of the regulation of responses to nutrition. The Creb3 transcription factor family appears to have a unique regulatory role that links cellular secretory capacity with development, nutritional state, infection, and other stresses. This role in regulating individual secretory capacity genes could place this family of transcription factors at an important regulatory intersection mediating an animal’s responses to nutrients and other environmental challenges. Interestingly, in both humans and mice, individuals with mutations in Creb3L3/CrebH, one of the Creb3 family members, exhibit hypertriglyceridemia (HTG) thus linking this transcription factor to lipid metabolism. We are beginning to understand how Creb3L3 and related family members are regulated and to dissect the potential redundancy and cross talk between distinct family members, thereby mediating both healthy and pathological responses to the environment. Here, we review the current knowledge on the regulation of Creb3 family transcription factor activity, their target genes, and their role in metabolic disease.

## Introduction

The recent rise in obesity and the chronic diseases associated with it are a result of many factors, including increased consumption of sugars and fats. Raising awareness of the potential adverse effects of these products in order to reduce their consumption has been a challenging task for many reasons. At the level of biomedical research, efforts are under way to identify possible therapeutic targets for nutritional disorders associated with our modern lifestyles. New strategies in the intervention of metabolic diseases are crucial to human health and will likely become ever important in the future.

The Creb3 family of transcription factors plays an interesting, but not yet fully understood role in regulating the secretory capacity of cells in response to environmental challenges. The founding member of the Creb3 family is the *Drosophila* CrebA protein, which was identified as binding to cyclic-AMP responsive DNA elements (CRE) (Abel et al., 1992; Smolik et al., 1992). Later, CrebA and the mammalian Creb3 family member, Creb3/Luman, was identified by its interaction with the host cell factor (HCF) (Freiman and Herr, 1997; Lu et al., 1998). Creb3, along with its other transcription family members, were implicated in endoplasmic reticulum (ER) stress responses (DenBoer et al., 2005; Asada et al., 2011). More recently, family members have been implicated in metabolism (Chin et al., 2005; Lee et al., 2010, 2011; Zhang et al., 2012; Kim et al., 2017a). Because of the high identity between the Creb3 family members, their shared function regulating secretory capacity and the overlap in their expression, understanding the potential for the cross talk and redundancy between family members will aid our understanding of the role that these transcription factors play in both healthy and unhealthy responses to nutrition and cellular metabolism.

## Creb3 Proteins Share Evolutionarily Conserved Domains

**Figure 1:**
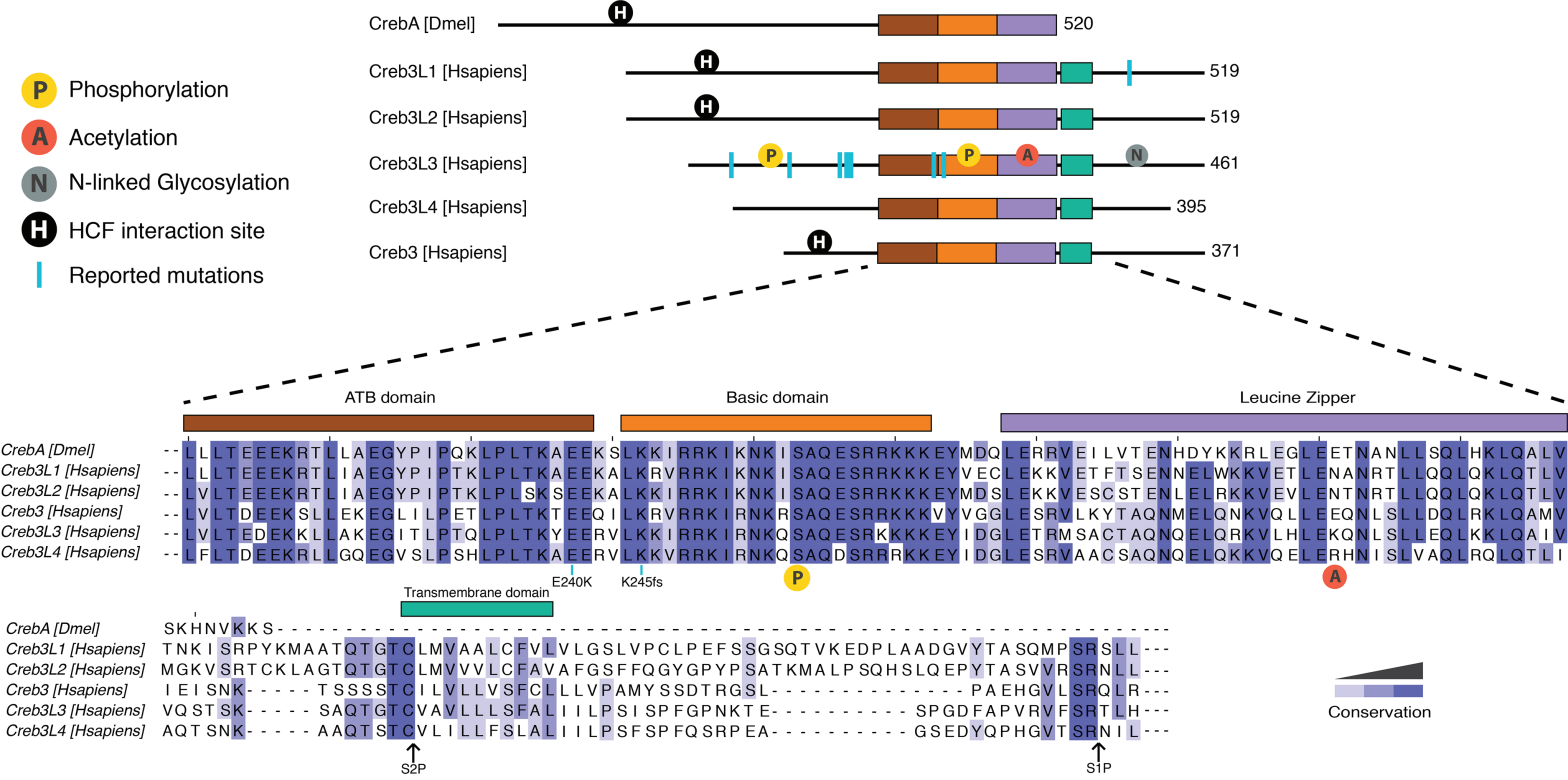
Protein organization of human and *Drosophila* Creb3 transcription factor family. All proteins share the highly conserved adjacent to bZip (ATB) (shown in brown), basic (orange), and leucine zipper (purple) domains. CrebA lacks the transmembrane domain (green) that allows the human Creb3 proteins to dock on to the ER and subsequently get cleaved by serine proteases at the S2P and S1P sites. The N-terminal fragment is then imported into the nucleus, where it binds DNA. CrebA, Creb3L1, Creb3L2, and Creb3 contain an interaction site for HCF (shown in black circle). Creb3L3 is modified by a number of posttranslational modifications, including phosphorylation (yellow circle), acetylation (red circle), and N-linked glycosylation (gray circle). Phosphorylation in the basic domain (yellow circle shown under the sequences) is conserved in all proteins. Reported mutations for family members are marked by blue lines. A mutation found in human Creb3L1 is Tyr428 which leads to a premature stop codon. Reported mutations in Creb3L3 are W46X, G105R, P166L, V180M, D182N, E240K, and K245fs. K245fs is a complex mutation found in three unrelated individuals. The mutation includes an insertion of a G in the first nucleotide of the codon 245 with another A to T mutation in the codon 247 resulting a frame shift mutation leading to a stop codon at codon 374. Mutations E240K and 245fs are conserved throughout human Creb3 proteins and CrebA. Sequence alignment was performed using Clustal Omega.

Mammals have five Creb3 family members, including Creb3/Luman, Creb3L1/OASIS (old astrocyte specifically induced substance), Creb3L2/BBF2H7, Creb3L3/CrebH, and Creb3L4/AlbZIP/Atce1/Tisp40/Creb4. For the purpose of a standard nomenclature, we refer to these proteins by their Creb3 names. Creb3 transcription factors are highly conserved from sponges to humans (Barbosa et al., 2013). One of their structural features and shared DNA binding domain is the leucine zipper, a dimerization domain that allows these proteins to act as homodimers and/or heterodimers (Figure 1; Zhang et al., 2006; Cui et al., 2016). The basic DNA-binding domains of the distinct family members are almost identical to each other, which suggests that they likely recognize the same DNA sequence. All mammalian Creb3 and CrebA proteins bind CRE and B-boxes (Abel et al., 1992; Lu et al., 1997; Omori et al., 2002; Chin et al., 2005; DenBoer et al., 2005; Kondo et al., 2005; Nagamori et al., 2006). Additionally, mammalian Creb3 proteins can drive the expression of an identical set of target genes in *Drosophila* embryos (Fox et al., 2010; Barbosa et al., 2013), which suggests an evolutionary conserved role across all family members. Furthermore, identical phenotypes are observed in knockout studies of the corresponding individual Creb3 homologs in fish and mice, such as the Creb3L2 gene (Melville et al., 2011; Hino et al., 2014b; Ishikawa et al., 2017). Together, these reports highlight a high level of functional and structural conversation, as well as possible redundancy and/or cross talk, between the distinct Creb3 family among different species.

In addition to the core DNA-binding domain, mammalian Creb3 proteins contain a transmembrane domain located C-terminally to the basic leucine zipper domain (bZIP), which allows it to anchor in the ER. Transcriptional activation then requires that the anchoring be removed *via* peptide cleavage through regulated intramembrane proteolysis, which releases the N-terminus of the transcription factor to shuttle into the nucleus. The cleavage process of the transmembrane domain is similar to the transcription factors ATF6 and sterol regulatory element-binding protein (SREBP) (Lemberg, 2011), another metabolically regulated transcription factor. An ER localization signal is located at the C-terminus of the bZIP (Llarena et al., 2010). The luminal domains are not very well conserved between family members, which may indicate that different mechanisms could regulate the cleavage of individual Creb3 family members.

All Creb3 proteins also share a unique adjacent to bZip (ATB) domain, which is located N-terminally to the bZIP (Figure 1). The ATB is necessary for all Creb3 protein’s transcriptional activity (Barbosa et al., 2013). However, how this domain activates transcription is not known. All members, except for Creb3L3 and Creb3L4, also have a linear motif that interacts with the co-activator HCF (Figure 1; Lu et al., 1998; Misra et al., 2005). Much more is known on how this linear motif mediates transcriptional activity compared to the ATB domain and we shall return to this point later in this review.

## Creb3 Proteins Regulate Biological Processes Requiring Protein Secretion

The transcriptional activity of Creb3 family members responds dynamically to various environmental cues and during various times in the life of an organism. Genetic studies on the mammalian Creb3 genes have focused on their developmental roles. Many of these studies suggest that this transcription factor family regulates protein secretion through the transcriptional of regulation of genes involved in COPII vesicle formation. Recently, studies indicate that several family members also have possible roles in responding to nutrients.

Both Creb3L1 and Creb3L2 have roles in development. During skeletal development, they regulate secretion of matrix proteins by the transcriptional regulation of the secreted protein, type 1 collagen (Col1a1), and secretory machinery genes involved in COPII vesicle formation (Murakami et al., 2009; Saito et al., 2009; Hino et al., 2014b). In humans, mutations of Creb3L1 results in improper and brittle bone development (Lindahl et al., 2018). In the intestine, Creb3L1 is responsible for the development of goblet cells (Asada et al., 2012), which secrete mucins into the lower digestive tract. Creb3L1 knockout mice exhibit an increased inflammatory response, potentially due to the lack of a protective mucus (Hino et al., 2014a).

Creb3L4, by contrast, is linked to chromatin organization during spermiogenesis (Adham et al., 2005; El-Alfy et al., 2006; Nagamori et al., 2006) and prostate cancer (Qi et al., 2002; Kim et al., 2017b), but recently, it has been linked to adipocyte function (Kim et al., 2014, 2015b). Intriguingly, Creb3L4 knockout mice only gain weight on a low-fat diet and not on a high-fat diet. This increase in weight gain does not appear to be due to a change in the amount eaten, but due to an increased adipocyte size. By contrast, mutants on a high-fat diet have smaller adipocytes. Additionally, blood glucose levels in the knockout animals are downregulated on a high-fat diet and higher on low-fat diet compared with wild type. It is not clear if these phenotypes are caused by Creb3L4 function in adipocytes or some other tissue and what, if any, role secretory capacity has in these phenotypes.

Originally, Creb3L3 was thought to have roles in liver development. However, Creb3L3 knockout mice develop normally (Luebke-Wheeler et al., 2008). Recent studies associate Creb3L3 with hypertriglyceridemia (HTG), lipid metabolism, and inflammation. Both humans and mice with Creb3L3 gene mutations, which result in a non-functional protein, have HTG (Figure 1; Johansen and Hegele, 2011; Lee et al., 2011; Zhang et al., 2012; Cefalu et al., 2015). Two human mutations associated with HTG (E240K, located in the ATB domain and K245fs located in the basic domain) have no transcriptional activity at Creb3L3-dependent promoters (Lee et al., 2011). Two additional human missense mutations associated with HTG, V180M, and D182N, have reduced transcriptional activity, but do not map to a known functional domain. Liver-specific knockout Creb3L3 mice also have HTG, corroborating hepatic role of Creb3L3 in regulating plasma triglyceride levels. Recent studies indicate intestinal Creb3L3 also regulates lipid metabolism, but particularly cholesterol metabolism (Kikuchi et al., 2016; Zeituni et al., 2016). In response to hepatitis C virus infections, Creb3L3 enhances TGF-β2 transcription, which increases fibrogenic responses in hepatic stellate cells (Chida et al., 2017). These results point to Creb3L3 regulating lipid metabolism and mediating responses to infection that in turn can result in inflammation.

Recently, genome-wide association studies strongly associate mutations in mice deficient for the recruitment factor of Creb3 (CREBRF) with increased body mass index and obesity (Loos, 2016; Minster et al., 2016; Naka et al., 2017). Originally, CREBRF was identified as a negative regulator of Creb3 through its ability to directly bind Creb3 (Audas et al., 2008). One of these human mutations, R457Q, maps to the region of CREBRF that interacts with Creb3. Interestingly, mice deficient for Creb3 and mice deficient for CREBRF have similar phenotypes. Both mutant mice suffer from low corticosterone, low body weight, and decreased pup survival due to reduced maternal care (Martyn et al., 2012; Penney et al., 2017), further suggesting that they interact in the same pathways. It would be interesting to determine which part of Creb3 interacts with CREBRF, whether other Creb3 family members bind CREBRF and whether mutations at the interaction site in these members are associated with metabolic diseases.

## Creb3 Transcription Factors Target Metabolic and Secretory-Machinery Genes

Genome-wide RNA expression studies indicate that the Creb3 transcription factor family can regulate evolutionarily conserved secretory machinery in a variety of systems (Lord et al., 2013; Dudek et al., 2015). Microarray analysis on human pancreatic beta-cells, as well as in HeLa cells overexpressing Creb3L1, shows upregulation of genes involved in extracellular matrix production and protein transport, such as KDEL-R3, Copγ2 (Vellanki et al., 2010), and other secretory machinery genes (Fox et al., 2010). The secretory genes regulated by Creb3L1 are comparable to the genes regulated by the *Drosophila* Creb3 member CrebA in the fly embryo (Fox et al., 2010). Creb3L2-knockout Medaka fish show a skeletal development phenotype and have reduced expression of genes involved in COPII vesicle formation (Ishikawa et al., 2017). In addition, transcriptional profiling studies from prostate cells highlight the involvement of Creb3L4 in the regulation of gene groups including protein sorting, maturation, and degradation (Ben Aicha et al., 2007).

To determine whether the Creb3 family of transcription factors may regulate these secretory machinery genes directly, several studies have used different techniques. CrebA has been probed to test whether it directly binds to the promoters of individual secretory machinery genes, specifically the signal peptide receptor α, sec61ß, spase25, p24.1, and Copζ (Fox et al., 2010). In mouse chondrogenesis, chromatin immunoprecipitation (ChIP) studies indicate Creb3L2 binds directly to the promoters of Sec23α and Sec24δ (Kondo et al., 2007). Recently, Creb3, Creb3L1, and Creb3L2 were also shown to regulate ARF4 and Copβ1 promoter activity (Howley et al., 2018). These studies indicate an interesting connection between Creb3 and the potential to regulate protein secretion. There is emerging evidence indicating that several mammalian Creb3 family members directly regulate COPII vesicle genes, suggesting that the Creb3 family members regulate the same target genes. Nonetheless, the *Drosophila* studies suggest that there are other secretory genes that are likely to be regulated by the mammalian family members in the mammalian context. The field needs genome-wide chromatin-binding data of the different family members to better understand the role of these family members in regulating secretory capacity.

In terms of family members directly regulating metabolism, Creb3L3, in particular, also directly regulates metabolic genes. Creb3L3 binds to metabolic genes involved in gluconeogenesis (Chin et al., 2005; Lee et al., 2010; Kim et al., 2017a) and genes encoding enzymes involved in triacylglycerol synthesis and fatty acid elongation (Zhang et al., 2012). In addition, Creb3L3 regulates the genes encoding proteins involved in lipid storage and transport such as the fat-specific protein 27 (Fsp27) (Xu et al., 2015) and the secreted proteins ApoC2 and ApoA4 (Zhang et al., 2012; Xu et al., 2014; Dandekar et al., 2016).

Clearly, more genome-wide chromatin-binding studies will allow the field to systematically study the role of how and under what nutritional conditions these transcription factors regulate the secretory machinery genes as well as other metabolic genes in order to establish an integrated and dynamic view of the transcriptional circuits underlying these important physiological responses to nutrients. These studies, complimented with reporters of transcriptional activity, will determine more concretely the role of this family in directly regulating the secretory machinery.

## Nutrient and Stress Signals Regulate Creb3 Family Transcript Levels

Most studies suggest that this transcription factor family is regulated primarily at the transcriptional level. ER stress, the best studied inducer of Creb3 family transcriptional activity, induces Creb3 family transcript levels (Kondo et al., 2005, 2007; Vellanki et al., 2010; Jang et al., 2011; Shin et al., 2012). However, this is not the case in all cell types (Vellanki et al., 2013). Some cytokines appear to regulate the levels of mRNA of these transcription factors (Jang et al., 2011; Shin et al., 2012), but not in all cell types (Kim et al., 2018). Inflammatory signals, such as TNFα, bacterial challenges, and hepatitis C virus (HCV) induce Creb3L3 gene transcription (Jang et al., 2015; Dandekar et al., 2016; Song et al., 2017; Troha et al., 2018). Which transcription factors mediate these proinflammatory signals is not clear. One candidate is the estrogen-related receptor-γ, which has been shown to mediate ER stress induction of Creb3L3 mRNA by directly binding and regulating the Creb3L3 gene (Misra et al., 2014).

The metabolic regulation of Creb3L3 gene activity is intriguingly complicated. Metabolic signals regulate Creb3L3 gene transcription in the liver and small intestine. In the liver, fasting and high-fat diet increases its transcript levels (Danno et al., 2010; Lee et al., 2010; Zhang et al., 2012; Vecchi et al., 2014; Xu et al., 2014). This fatty acid induction of Creb3L3 mRNA is repressed by insulin (Danno et al., 2010; Gentile et al., 2010). Additionally, transcript levels are regulated in a circadian manner (Zheng et al., 2016). Fewer studies have looked at Creb3L3 levels in other tissues, but a recent study indicates that a high-fat diet also induces Creb3L3 transcript in the zebrafish intestine (Zeituni et al., 2016). Studies indicate that peroxisome proliferator-activated receptor alpha (PPARα) may regulate Creb3L3 gene activity in response to fasting and to high-fat diets in the liver (Kersten et al., 1999; Danno et al., 2010). Another transcription factor that may regulate Creb3L3 in response to inflammatory signals (Babeu and Boudreau, 2014) and lipid metabolism in the small intestine (Carriere et al., 2005), is the hepatocyte nuclear factor 4 alpha (HNF4-α), which also binds to the promoter region of Creb3L3 to regulate its expression in the liver (Luebke-Wheeler et al., 2008). Clearly, Creb3L3 gene activity is sensitive to metabolic cues and will be regulated by additional factors.

Considering that Creb3 proteins must undergo proteolytic cleavage from the ER to be active, it is not completely clear, however, whether and how transcriptional and posttranscriptional regulation may be coupled. It appears that the proteolytic cleavage may be constitutive and also that generally Creb3 proteins may be quickly turned over (Bailey et al., 2007). Therefore, transcriptional control appears to be an important regulatory step for the activity of these proteins.

## The Creb3 Family is Regulated at the Protein Level by Intramembrane Proteolysis

Surprisingly, proteolytic cleavage of the well-conserved ER anchoring domain releasing the active form seems to be generally constitutive. In most studies in which both the transcript and the protein is monitored, the transcript levels seem to be limiting, thereby suggesting that transcription or RNA degradation is the key mechanism of regulation for this family of proteins. However, there are circumstances that Creb3L3 intramembrane proteolysis appears to be regulated. Creb3L3 levels and activity change throughout the day, but the levels of the RNA transcript and of the C-terminal cleaved protein show different oscillations. The circadian oscillations of the cleaved C-terminal Creb3L3 increase in the late day, when the transcript and uncleaved protein are the lowest (Zheng et al., 2016; Kim et al., 2017a). This circadian regulation of the intermembrane cleavage is controlled by the core clock component BMAL1 and the AKT/GSK3β signaling pathway (Kim et al., 2017a). Another example is during osteclastogenesis, where the cytokine RANKL induces higher levels of the cleaved C-terminal Creb3L3 without induction of the transcript, in contrast to IL-1 during osteclastogenesis, which induces the transcript, but does not lead to the increase in the cleaved form (Kim et al., 2018). A better understanding of the Creb3 proteolytic cleavage regulation will come from studying these more biologically relevant stimuli. However, current data already indicate that there likely are physiological processes that are important in the fine tuning of Creb3L3 transcription factor activity.

## Posttranslational Control of Creb3 Proteins

Several posttranslational modifications have been observed on Creb3 that affect transcriptional activity. Phosphorylation was shown to both regulate intramembrane proteolysis (Zheng et al., 2016) and Creb3L3 protein turnover (Barbosa et al., 2015, 2017; Cheng et al., 2016). Glycogen synthase kinase 3 beta (GSK3β) and casein kinase II regulate Creb3L3 turnover *via* serine residues in the bZip domain (Figure 1; Barbosa et al., 2015; Cheng et al., 2016). In addition, GSKβ mediates the circadian-regulated intramembrane proteolysis of Creb3L3 *via* the phosphorylation of S260 (Figure 1; Zheng et al., 2016). In contrast to phosphorylation, which decreases Creb3L3 activation, N-linked glycosylation at N412, N420, and N427 are required for efficient intramembrane proteolysis (Chan et al., 2010). However, we have little biological and mechanistic insight into the regulation of Creb3L proteolysis *via* glycosylation. Acetylation of K294 of Creb3L3 appears to be regulated by several different stimuli. It transiently increases with fasting and glucagon, as well as in a circadian manner during the day, which enhances interactions with the transcription factor PPARα (Kim et al., 2015a, 2017a).

Currently, there is little evidence whether other members of the family are posttranslationally modified. However, given that family members share conserved domains, it is likely that they are regulated in a similar fashion. Further experiments will allow us to understand this better.

### Redundancy and Cross Talk Between Creb3 Protein Family Members

**Figure 2:**
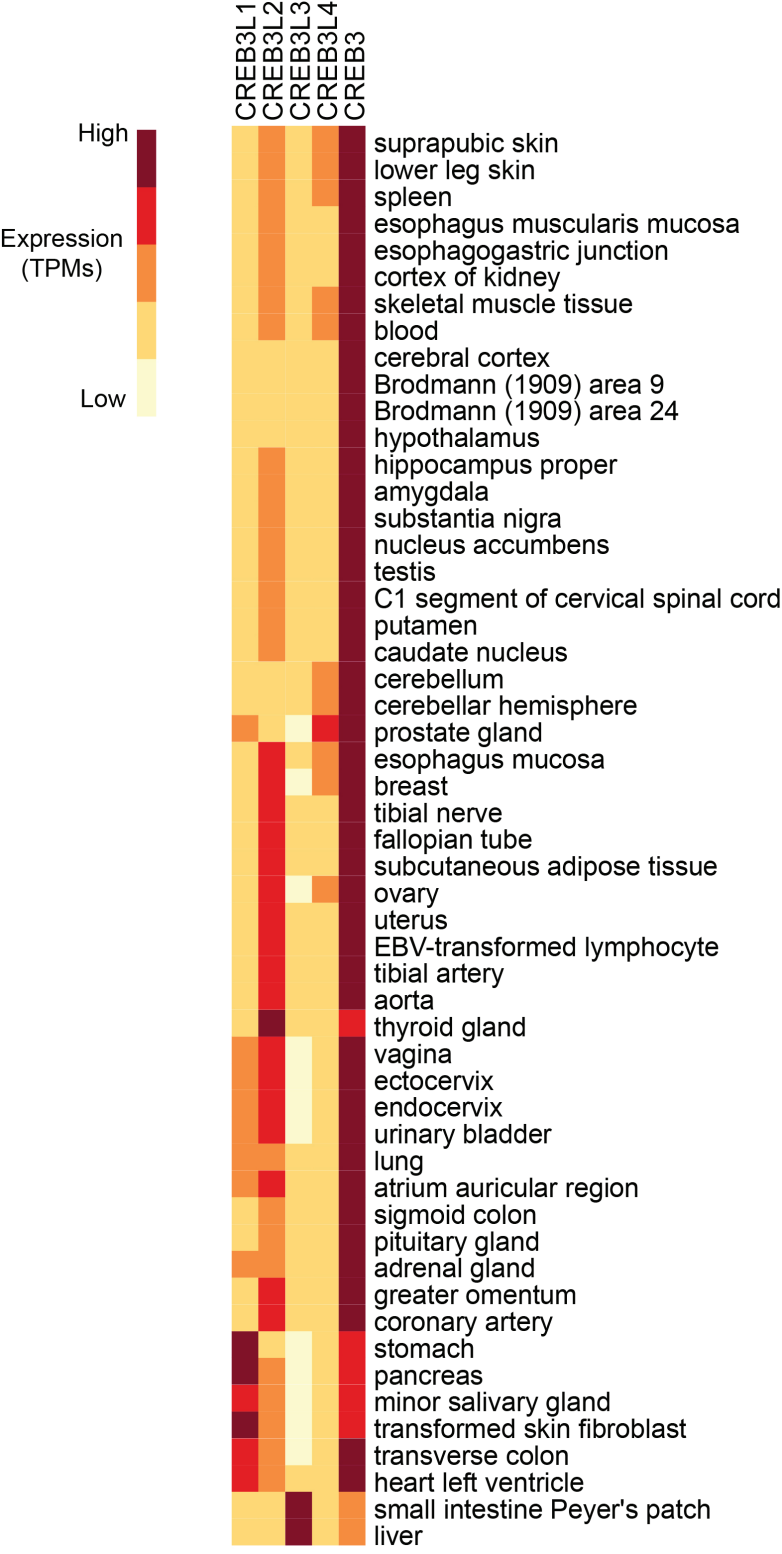
RNA expression analysis of Creb3 transcription factor family across 43 tissues from 175 individual humans (Consortium, 2015). Creb3 is highly expressed in almost all tissues. Creb3L1–4 transcripts are more tissue specific. Creb3L3 is predominantly expressed in prostate glands. Creb3L3 is highly expressed in the small intestine and liver, where it has been shown to regulate metabolism. TPM-normalized data were downloaded from the EMBL-EBI Expression Atlas database (www.ebi.ac.uk) and plotted using R.

While some family members are expressed in multiple tissues throughout the organism, some are more tissue specific (Figure 2). Different family members may compete for binding in the same cell type, implicating functional redundancy. Conversely, presence of the bZIP domain in all Creb3 proteins as well as overlapping expression patterns in tissues, suggest cross talk between family members. While Creb3 is highly expressed and the most ubiquitously expressed of the Creb3 family, other family members are more limited in their expression to specific cell types and are transiently induced in some tissues. For example, Creb3L3 is most highly expressed in the liver and the small intestine (Luebke-Wheeler et al., 2008), where it has been known to function. However, it is transiently expressed in osteoclastogenesis (Jang et al., 2011; Kim et al., 2018). When Creb3L3 is expressed in calvarial osteoblast cells, it blunts the cellular responses to bone morphogenetic protein 2 (BMP2) potentially by reducing Creb3L1 levels (Jang et al., 2011).

Creb3L family members heterodimerize with each other to regulate Creb3 proteins transcriptional activity, possibly through the regulation of intramembrane proteolysis. In astrocyte differentiation, Creb3L1–Creb3L4 dimerization correlates with activation of Gcm1 transcription (Cui et al., 2016). By contrast, Creb3L1-Creb3 dimerization leads to the downregulation of Gcm1transcription. Creb3L4 is shown to inhibit Creb3L1 activity in prostate cancer cells (Cui et al., 2016). In this study, they show that these Creb3 proteins interact *via* their bZIP domain. This interaction inhibits the release of the active N-terminus of Creb3L1 from the ER. Clearly, studying the interactions between Creb3 family members has begun to reveal the role of cross talk or redundancy between the distinct family members.

## Creb3 Protein-Protein Interactions Regulating Chromatin

Here, we will only focus on the Creb3 family protein-protein interactors that occur in the nucleus and that can directly regulate transcription and chromatin function. As discussed previously, most Creb3 proteins contain a linear motif which allows them to interact with HCF. HCF, in turn, recruits chromatin factors, such as histone K4 H3 methyltransferase enzymes (Set1 and MLL) (Wysocka and Herr, 2003), a demethylase (LSD1) (Liang et al., 2009), acetyltransferases (ATAC/STAGA, MOF) (Dou et al., 2005; Smith et al., 2005; Guelman et al., 2006), and a deacetylase (sin3a/HDAC) (Wysocka and Herr, 2003). These interactions suggest the type of chromatin changes that Creb3 family members activity may regulate. HCF is particularly interesting in metabolism, since it is a transcriptional regulator of cell cycle that is also cleaved by the nutrient-responsive glycosyltransferase, O-linked N-acetylglucosamine (O-GlcNAc) transferase (OGT) (Capotosti et al., 2011). OGT mediates the addition of a sugar moiety, O-linked β-N-acetylglucosamine (O-GlcNAc) in response to diverse nutritional and hormonal cues and is implicated in many metabolic disorders such as diabetes and cardiovascular disease (Ruan et al., 2013).

Another possible co-activator of Creb3 proteins is the CREB-regulated transcriptional coactivator 2 (CRTC2 or TORC2). Just like HCF, it is also implicated in metabolic responses (Koo et al., 2005; Wang et al., 2009). Based on a recent study (Lee et al., 2010), CRTC2 interacts with Creb3L3 to induce transcription.

## Outlook

The available data indicate that Creb3 family members lie at a unique regulatory node receiving developmental and environmental signals, both metabolic and inflammatory, to regulate gene expression, including secretory capacity. Creb3 proteins can regulate genes involved in COPII vesicle formation by directly binding to their promoters. Although Creb3L3 regulation is the best studied in response to nutrition and metabolic disease, studies of other family members, such as Creb3 and Creb3L4, suggest that they also have roles in responding to nutrient status.

While Creb3 family members’ potential regulation of secretory capacity is an interesting mechanism to couple nutrient status to a system-wide physiological response, we currently do not have a complete understanding of the secretory genes Creb3 family transcriptionally regulate. Most of the genome-wide data comes from RNA studies, while ChIP studies have generally focused on a few target genes. The mRNA-seq studies have been very useful in identifying a list of putative Creb3 family target genes. Several ChIP or transcriptional reporter studies have looked at individual or a combination of putative targets. There are antibodies available for most of the family members which have been used for immunoprecipitations and ChIP. These antibodies should be used for ChIP-seq experiments. Genome-wide ChIP studies will aid our global understanding of which secretory machinery genes as well as other genes Creb3 transcription factors directly regulate. Knowing these direct targets will benefit our understanding of the physiological role that these transcription factors have in responding to metabolic challenges.

Furthermore, what is the physiological impact of regulating these secretory genes? Clearly, the export of hormones responding to the nutritional statues of an organ would impact inter-organ communication. Which hormones are impacted is an important question. However, changes in the secretory machinery could also alter lipid export and thus lipid metabolism, as well. In addition, changes in nutrition may also cause changes in the morphology of the ER and Golgi, as well as changes in membraneless cellular structures, such as processing bodies and stress granules (van Leeuwen et al., 2018), which are known to be regulated by nutrition. Is it possible that changes in secretory proteins also contribute to the regulation of these interesting cellular structures? In sum, by regulating secretory machinery genes individually or systematically, which remains to be established, Creb3 family members will likely have roles both at the cellular level and at the system-wide, inter-organ level. More biologically relevant model systems will need to be used than has currently been possible, such as tissue-specific and conditional knockdowns of specific family members within a living, adult organism. Such studies will be useful to understand the redundancy and cross talk between family members. In addition, these studies will also be useful to better understand the physiological role Creb3 proteins play in the regulation of the secretory machinery particularly in response to nutrients and under healthy or pathophysiological conditions.

## Author Contributions

Both authors contributed equally toward this work.

### Conflict of Interest Statement

The authors declare that the research was conducted in the absence of any commercial or financial relationships that could be construed as a potential conflict of interest.
